# Supplementary material for: Revisiting stress-corrosion cracking and hydrogen embrittlement in 7xxx-Al alloys at the near-atomic-scale
Source: Nat Commun. 2022 Jul 25;13:4290. doi: 10.1038/s41467-022-31964-3 (PMC9314352; doi:10.1038/s41467-022-31964-3)
Supplement: Supplementary file 1 — Supplementary Information [file 41467_2022_31964_MOESM1_ESM.pdf]

Supplementary Information for

Revisiting stress-corrosion cracking and hydrogen embrittlement in 7xxx-Al alloys at the near-atomic-scale

*Martí López Freixes, Xuyang Zhou, Huan Zhao, Hélène Godin, Lionel Peguet, Timothy Warner, Baptiste Gault**

** corresponding authors. E-mail addresses: b.gault@mpie.de*

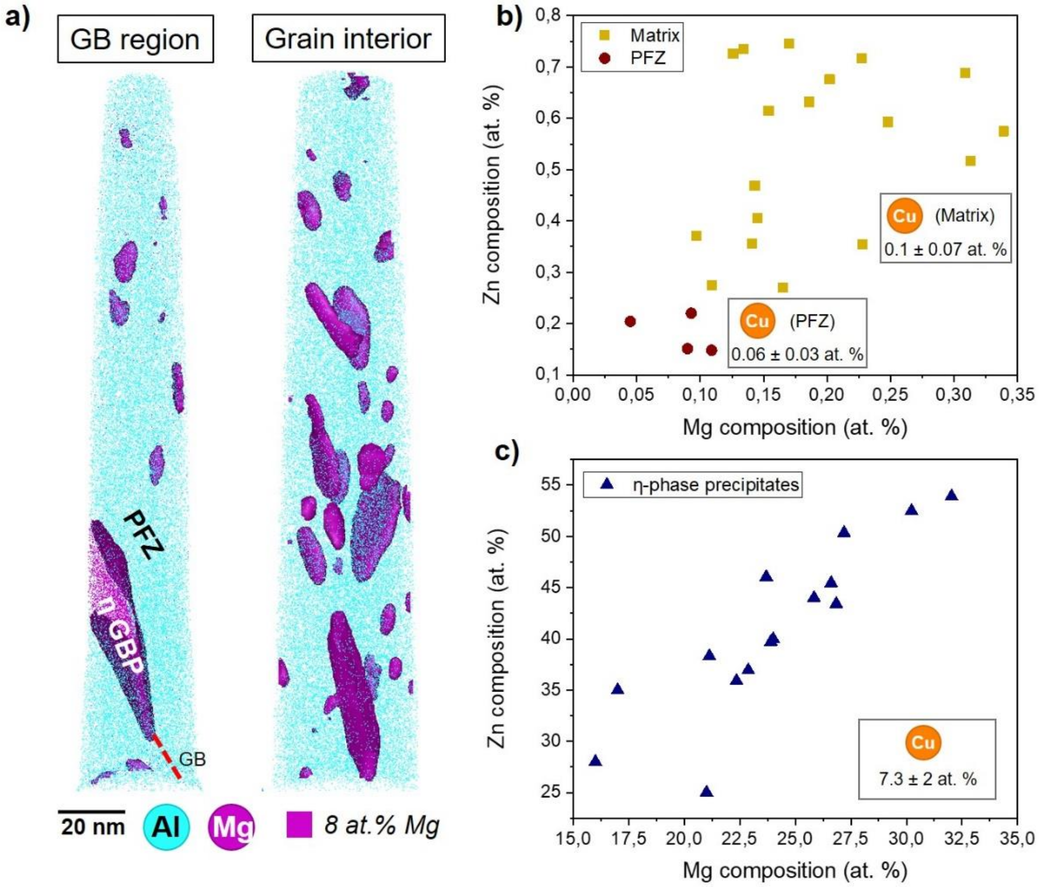

Supplementary Figure 1. Sample characterization far from the crack. a) APT reconstruction of a GB and a grain interior from an undeformed region; b) Solute matrix composition from 4 datasets and PFZ reference compositions from the dataset shown above; c) η-phase precipitates reference compositions taken from the grain interior from 3 datasets and the near GB region from the dataset shown above. Only plate shaped precipitates were analysed. The error values reported for Cu correspond to the standard deviation of all measurements.

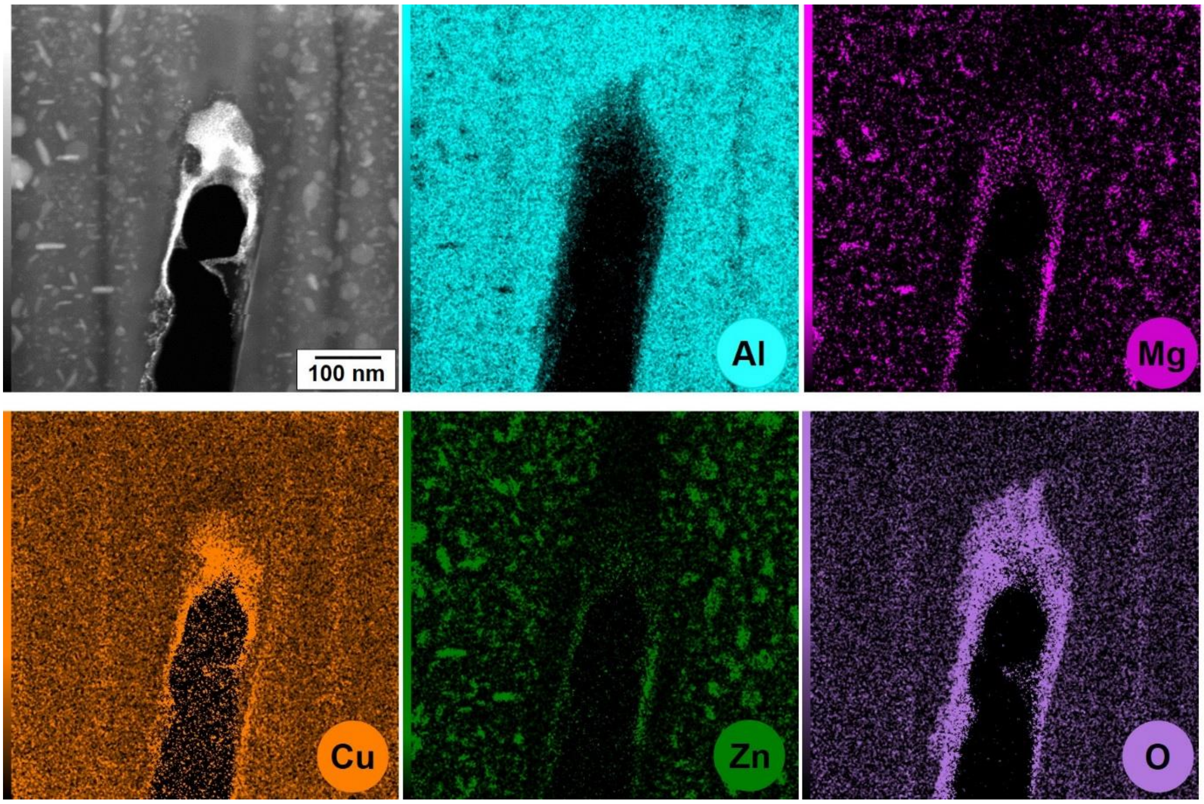

Supplementary Figure 2. Compositional characterization of the crack region. Composition maps of the stress-corrosion crack shown in Figure 1b.

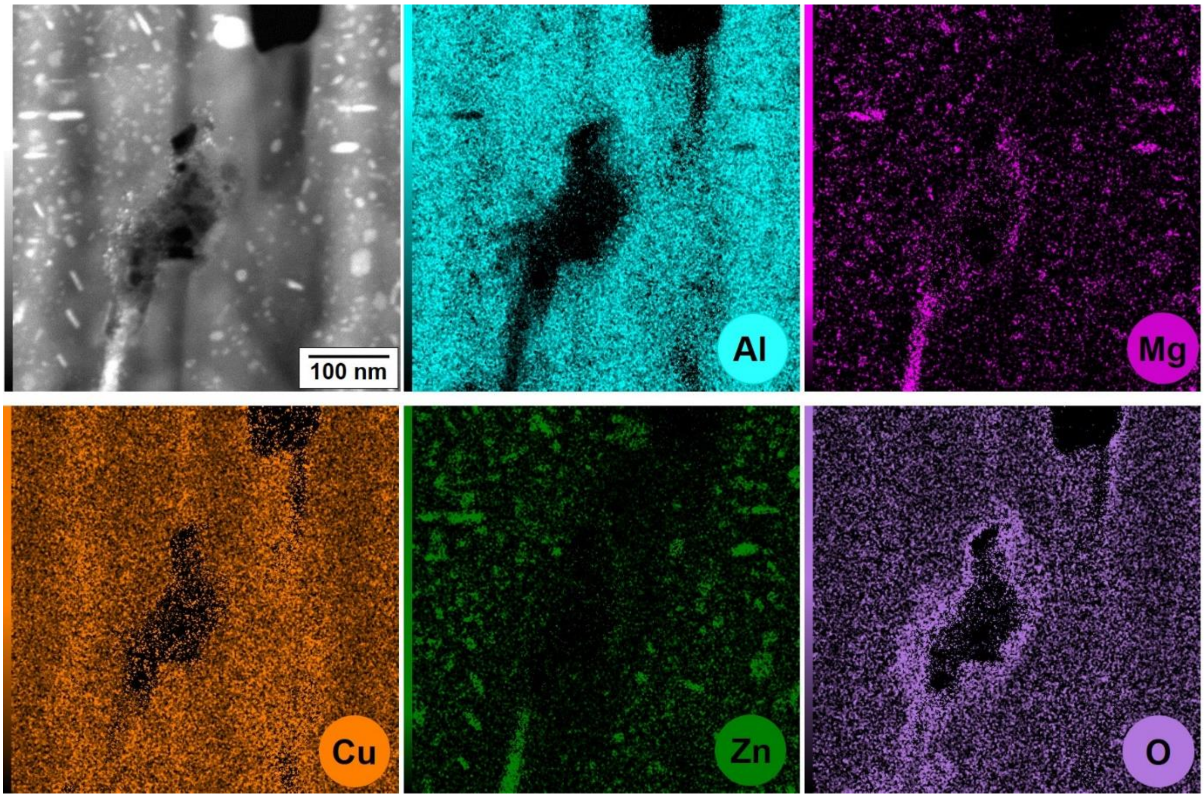

Supplementary Figure 3. Compositional characterization of the void-like region below the crack. Composition maps of the void-like features shown in Figure 1c.

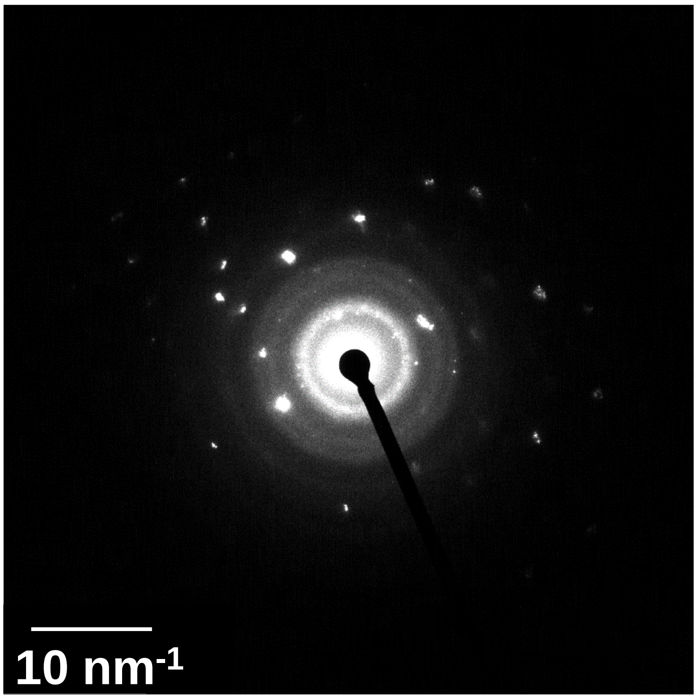

Supplementary Figure 4. Crystallographic characterization of the oxide at the stress-corrosion crack. Selected area electron diffraction pattern from the oxide region in Figure 1a.

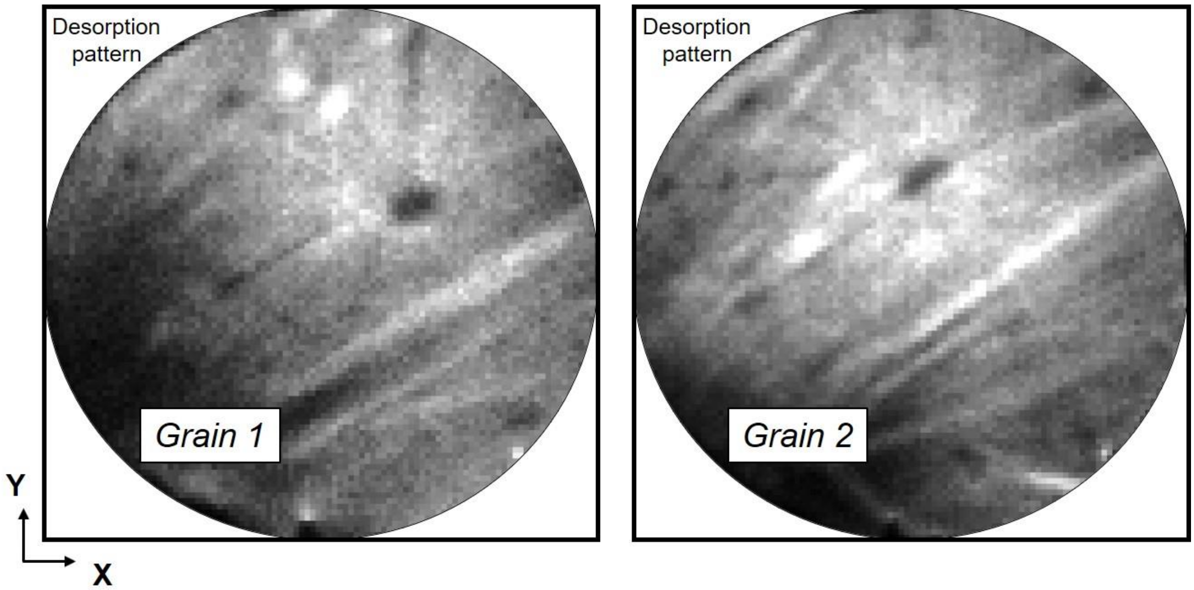

Supplementary Figure 5. Stress-corrosion crack intergranular propagation. Desorption pattern showing two distinct crystallographic orientations within the crack tip (Figure 2b)

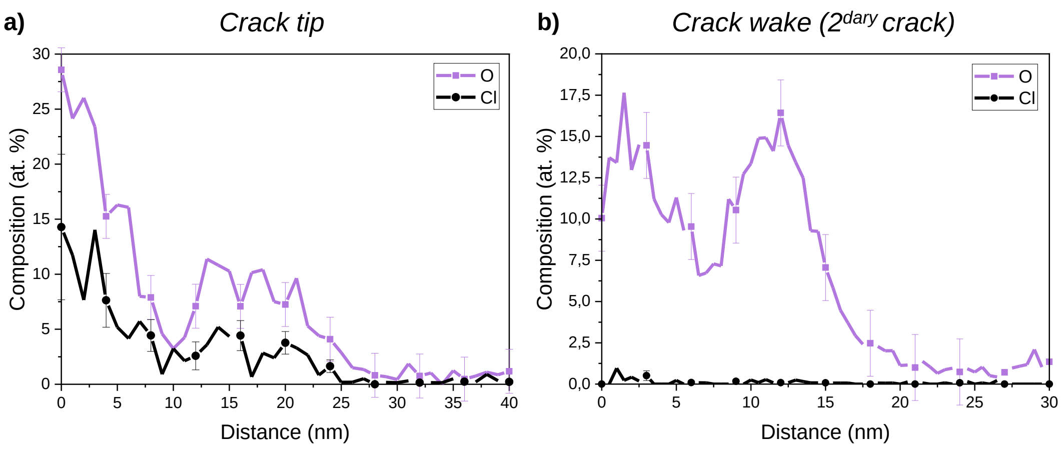

Supplementary Figure 6. Cl incorporation in the oxide film at different crack locations. Cl composition in the oxide at the crack tip and at the 2ndary crack. Oxygen profiles replotted from Figure 2c and g. The error bars correspond to the standard deviation within each of the bins in the profile.

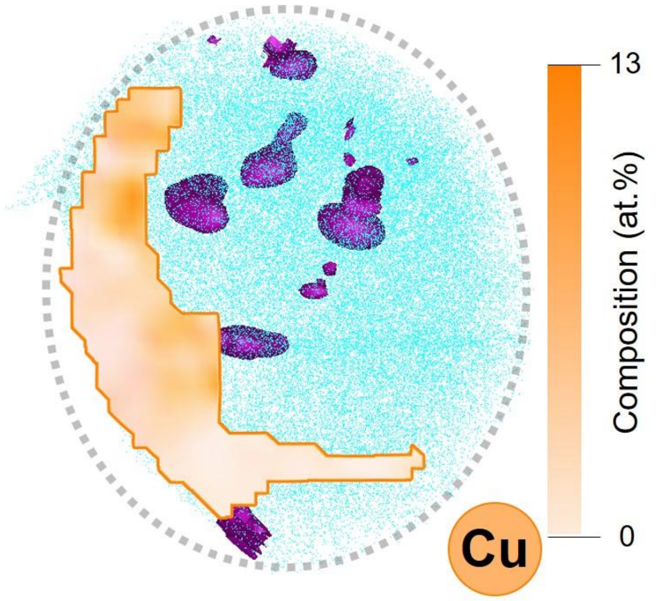

Supplementary Figure 7. Cu enrichment at the crack tip oxide/metal interface. 2D Cu composition map in the oxide at the crack tip. The map was obtained from a 10 nm slice shown in Figure 2b

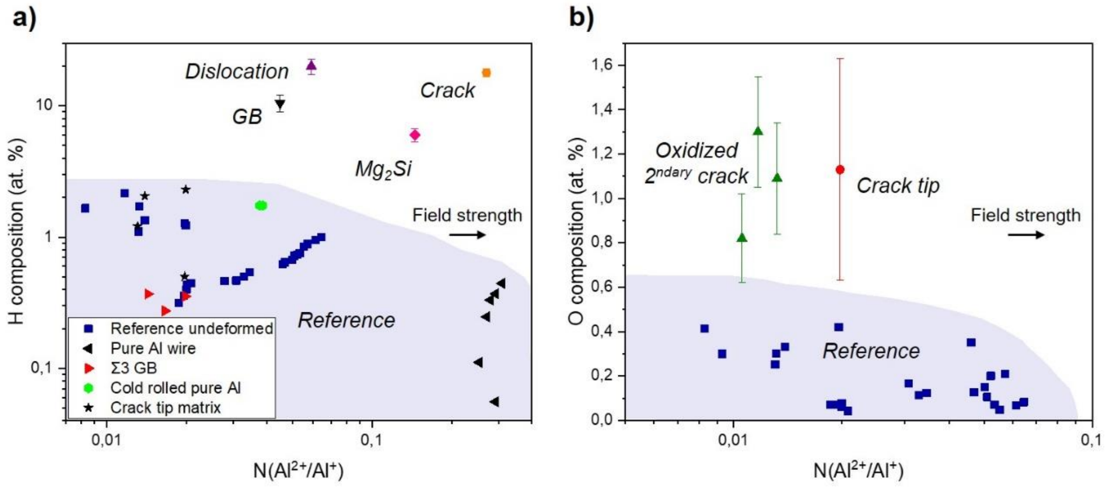

Supplementary Figure 8. Reliabilty of H/O measurements in APT. a) Hydrogen composition within the oxide in the secondary crack (Figure 2f) and at the dislocation, the Mg_2Si particle and the GB (Figure 1e) plotted against field strength during the APT experiments, showing an increase with respect to reference values at similar field strengths. Reference values include measurements on undeformed material, drawn and cold-rolled pure Al, and a Σ3 GB in an Al bi-crystal. b) Oxygen composition in the matrix adjacent to the crack tip (Figure 2b) and the oxidized 2ndary crack (Figure 2f), also showing increased levels. Values from the matrix from several datasets are included as reference. The error bars correspond to the standard deviation within each measurement.

.
